# Use of complementary and alternative medicine by patients with hypermobile Ehlers–Danlos Syndrome: A qualitative study

**DOI:** 10.3389/fmed.2022.1056438

**Published:** 2022-12-14

**Authors:** Tom A. Doyle, Colin M. E. Halverson

**Affiliations:** ^1^Center for Bioethics, Indiana University School of Medicine, Indianapolis, IN, United States; ^2^Department of Medicine, Indiana University School of Medicine, Indianapolis, IN, United States; ^3^Department of Anthropology, Indiana University, Indianapolis, IN, United States; ^4^Regenstrief Institute, Indianapolis, IN, United States; ^5^Charles Warren Fairbanks Center for Medical Ethics, Indianapolis, IN, United States

**Keywords:** Ehlers–Danlos Syndrome, complementary therapies, alternative medicine, holistic health, homeopathy, integrative medicine, qualitative interviews

## Abstract

**Background:**

Patients with hypermobile Ehlers–Danlos Syndrome (hEDS) often make use of complementary and alternative medical (CAM) techniques to manage their chronic pain and other symptoms. Nevertheless, how they use CAM, which techniques they favor, and how CAM use affects their allopathic care remain unclear. The purpose of this qualitative study was to understand patients’ personal experiences with CAM and its role in their symptom management.

**Materials and methods:**

Thirty individuals living with hEDS completed a brief online survey related to their CAM use. Thereafter, in-depth interviews were conducted with 24 of the survey respondents, qualitatively investigating their experiences with CAM. Data were analyzed using thematic analysis.

**Results:**

Participants described massage therapy (*N* = 21), medical cannabis (*N* = 12), and mindfulness (*N* = 13) as some of the most useful CAM modalities for managing symptoms related to hEDS, but they expressed a general interest in pursuing any treatment that could potentially reduce their chronic pain. They suggested an overall trust in CAM modalities and practitioners and ascribed greater empathy to CAM practitioners than to conventional medical providers. However, they also described a critical skepticism of CAM (and conventional) therapies and recounted instances of injury from such treatments.

**Conclusion:**

Participants made extensive use of CAM therapies. They described both critical benefits as well as harms from the use of these non-conventional modalities. These results underscore the importance of clinicians maintaining communicative and compassionate relationships with their patients, and of an openness to the discussion and use of CAM treatments.

## 1 Introduction

Ehlers–Danlos Syndrome (EDS) is a connective tissue disorder with 13 types, of which hypermobile Ehlers–Danlos Syndrome (hEDS) is the most common. One of the primary features of hEDS is chronic pain, but it is also prominently characterized by joint hypermobility and fatigue. Chronic pain in particular—and its associated disability—is considered a difficult symptom to manage clinically, with conventional methods often having limited and impermanent effects (1), and opioids now considered incapable of successfully controlling it (2).

Many patients with hEDS therefore turn to complementary and alternative medicine (CAM) (3). While many CAM therapies may at first appear peripheral to or outside of standard American conceptions of health seeking behavior, a 2015 study found that around 40% of adults had nonetheless trialed a CAM modality at least once (4). These modalities are increasing in importance and popularity as adjuncts to conventional medical treatment and are now offered in many hospitals. In fact, in 2010, it was reported that 42% of US hospitals offered one or more CAM services (5), and 93% of Veterans Health Affairs medical centers offered at least two such services in 2015 (6). A 2016 study estimated that $30.2 billion is spent out of pocket per year on complementary and integrative therapies (7). Additionally, the National Center for Complementary and Integrative Health at the National Institute of Health has been supporting an increasing amount of scientific research and randomized controlled trials to test the efficacy of CAM modalities. Pertinent to the hEDS patient population, much of this research focuses on the use of CAM therapies in the treatment of chronic pain. Thus, a growing body of data exists on the use of yoga, meditation (for example, mindfulness), and physical therapy in the treatment of chronic pain (8, 9).

While CAM use is widespread, certain demographics more frequently use CAM therapies. People with chronic illnesses (10), in particular those with chronic pain (11), use CAM more frequently than do others. Additionally, women use these treatments at higher rates than do men (12). As hEDS is a chronic illness characterized by pain and it is estimated that more than 80% of those with a diagnosis are women (13, 14), the hEDS population is particularly likely to make use of CAM. However, little has been written on the topic [but see Demes et al. (3) and Dar (15)], and what has been published provides only a limited glimpse into the full extent and implications of this phenomenon. While prior literature has called on future studies to examine the role that accessibility has on which CAM therapies patients eventually trial (3), more basic questions persist alongside this issue. Specifically, the questions of what CAM modalities are being used and which are being favored by patients with hEDS remain unanswered, as do questions related to their trust in and skepticism of CAM. A more comprehensive understanding of CAM usage among the hEDS population may allow clinicians to anticipate patients’ CAM usage, to understand what modalities they may prefer, and to be aware of the potential injuries and harms that may arise for this population from certain CAM modalities. We therefore sought to investigate these fundamental questions qualitatively, along with the question of accessibility, through a brief survey and a series of in-depth interviews with individuals living with hEDS. The goals of our study were to determine (1) patients’ motivations in seeking CAM modalities, (2) which CAM modalities they commonly trial, (3) how they assess the usefulness of these specific therapies, and (4) their level of trust and skepticism in CAM practitioners and modalities.

## 2 Materials and methods

This study was approved by the Indiana University Institutional Review Board (protocol number 2003882072). All participants provided both written and verbal informed consent. All candidates were at least 18 years of age and spoke English. They were also patients in an EDS clinic and had an official, clinical diagnosis of hEDS that was made using the 2017 diagnostic criteria (16). Patients were notified of the project by a clinician at the EDS clinic. If an individual expressed interest, a researcher then attempted to contact them by phone up to three times. If a candidate agreed to participate, they were then sent a link to the survey. After participants had submitted the survey, a researcher contacted them again with an offer to participate in the interview portion of the study. Those who wished to continue their participation scheduled a mutually acceptable time for the phone interview. All participants were assigned a random, three-digit pseudonym.

### 2.1 CAM survey

All participants completed an initial exploratory survey designed to identify CAM use among our research participants using REDCap, a secure, web-based application used to manage online surveys and databases. The survey questionnaire was constructed based on a literature review and assessed their use and evaluation of various CAM modalities. Demographic data were also ascertained. In order to access the survey, participants were required to complete a written informed consent. Survey responses were collected during spring 2022.

### 2.2 CAM interviews

Between spring and summer 2022, a subset of these respondents was selected using convenience sampling and asked to complete a subsequent interview. The interview guide was constructed based on data from the initial surveys and a second literature review. These in-depth, semi-structured interviews covered participants’ conceptualization of CAM, their use of such modalities in their own care, the outcomes of those treatments, accessibility issues, and trust and skepticism in CAM and its practitioners. The interview guide is available upon request from the corresponding author. Thematic saturation was reached when no new insights emerged from additional data collection, and at this point recruitment of further participants was halted (17).

All interviews were conducted over the phone or *via* Zoom by a study team member (CMEH). Before the interview, participants completed an oral consent. The interviews were audio-recorded and transcribed professionally. Analysis was conducted by both authors, one (CMEH) who is a medical anthropologist and bioethicist and the other (TAD) who is a philosopher and bioethicist. Both have professional training in qualitative research methods. The senior author has worked extensively with this patient population, and it is the focus of his NIH-funded research program. The team used Dedoose, a mixed-methods analysis tool, to conduct thematic analysis in order to investigate the data qualitatively and systematically (18), following Braun and Clarke’s six steps of thematic analysis (19). First, they familiarized themselves with the data through an intensive review of the transcripts. They independently identified themes and established agreement on each theme. In order to explore the experiences and values of the participants and to ensure the quality and validity of the findings, researchers followed recognized standards for qualitative research (20). Initial themes were then reviewed and refined in iterative fashion as patterns in the data began to emerge and a comprehensive narrative of the data as a whole was generated. Quotations illustrating the main themes were identified during this process.

## 3 Results

A total of 30 unique individuals completed the survey (response rate: 97%). It took on average 5 min to respond to all the questions. Twenty-four of the survey respondents additionally completed an interview (response rate: 100%). The average length of an interview was 52 min (range: 20–89 min). All participants were residents of the United States. The majority identified as female (80% in the surveys, 83% in the interviews) and White (80% in the surveys, 96% in the interviews), and the average age of participants was just under 40 years. Relatively equal percentages of respondents described themselves as Christian, spiritual, followers of some other religion, or having no religion. A fairly uniform spread of educational attainment was also represented. For more information on participants’ demographics, see Table 1.

**TABLE 1:** Participant demographics.

		Survey: *N* = 30 (%)	Interviews: *N* = 24 (%)
Gender	Female	24 (80%)	20 (83%)
	Male	1 (3%)	1 (4%)
	Non-binary	4 (14%)	3 (13%)
Religion	Christian	10 (33%)	10 (42%)
	Spiritual	6 (20%)	5 (21%)
	None	8 (27%)	5 (21%)
	Other	6 (20%)	4 (17%)
Education	High school	5 (17%)	3 (13%)
	Some college	7 (23%)	5 (21%)
	Bachelor’s degree	10 (33%)	9 (38%)
	Graduate degree	8 (27%)	7 (29%)
Race/Ethnicity	Non-Hispanic white	27 (80%)	23 (96%)
	Hispanic	1 (3%)	1 (4%)
	Black	1 (3%)	0 (0%)
	More than one race	1 (3%)	0 (0%)
Age (years)	Average	39	38
	Range	18–62	18–62

Three themes emerged from our interviews as particularly salient to a deeper understanding of CAM use among patients with hEDS, namely, (a) motivators and demotivators for pursuing such therapy, (b) approval and disapproval of different modalities, and (c) trust and skepticism of CAM. Each of these themes is discussed in detail below, and further details concerning these themes can be found in Table 2.

**TABLE 2:** Major themes.

Themes	(Subthemes)	Representative quotes
Motivators and demotivators for CAM use	Desperation for relief	“There were definitely things at first where I was like, I’m just not going to do this. Then as time went on […] desperation rose” (321).
	Access and affordability	“Oh my God, this is so expensive! Maybe I don’t need it” (158).
Approval and disapproval of CAM modalities	Most useful CAM modalities	“I find that the best for me is topical versions of [cannabis] that are very helpful when I’m having muscle spasms and deep pain” (721).
	Least useful CAM modalities	“The chiropractor just made [my symptoms] worse, and acupuncture was very painful but didn’t help anyway” (418).
Trust in CAM modalities and practitioners	Relationship with CAM practitioners	“I think [CAM practitioners] are more open to listening to what you have to say and your story and background” (983).
	Disclosure of CAM use to medical providers	“I don’t openly always disclose the cannabis because […] you get a look. And then I know that will probably jade them for the rest of my treatment” (500).
	Skepticism and injury from CAM	“[The chiropractor] did a whole lot more damage than help [and I needed] years to recover from it” (983).

### 3.1 Motivators and demotivators

#### 3.1.1 Desperation for relief

Every one of the survey respondents had trialed at least one CAM modality. The average respondent had trialed nine different CAM modalities (range: 1–28). While a minority chose not to pursue specific therapies, the majority of participants described a “desperation” for relief from their symptoms—particularly, their chronic pain, but also anxiety, dizziness, and insomnia, among other things—as motivating their decision to pursue these treatments. “I don’t think […] anything’s been recommended for me that’s like, ‘No, definitely not!’” (400). They explained that conventional treatments were not sufficient in managing these issues. Participants also explained that as their desperation increased, their overall discomfort with CAM decreased: “There were definitely things at first where I was like, I’m just not going to do this. Then as time went on […] desperation rose,” and ultimately, this interviewee found no proffered treatment beyond her threshold of comfort (321).

#### 3.1.2 Access and affordability

Participants reported that very few CAM treatments were covered by their insurance. And many of the treatments that had been covered, were only covered under particular context- and time-limited conditions. For instance, some insurers initially paid for massage therapy in the immediate aftermath of a physical injury. However, as our participants’ conditions were chronic, once the allotted time had passed since the relevant injury, coverage was then stopped despite the continuation of their symptoms. Some CAM modalities, such as cupping and dry needling, were also covered when performed by a physical therapist, but the same services were not covered when performed by a chiropractor.

Participants were thus left to pay for most of their CAM treatments out of pocket. [Some participants were even so frustrated by the complexities of the reimbursement systems that they chose to pay out of pocket for less expensive services rather than “fight for it to be covered by insurance” (506).] Participants described their CAM regimens as a significant burden on their budgets. “It’s very expensive to be disabled,” one woman explained (403). They described the cost of CAM treatments as a major limiter to their ability and willingness to use them. “Oh my God,” one woman recalled, thinking of a particular treatment option, “this is so expensive! Maybe I don’t need it” (158). However, several individuals stated that they found specific CAM treatments to be critical to managing their symptoms. One woman reported that prolotherapy (a treatment involving the injection of an irritant solution into a joint) greatly improved her quality of life: “I had [prolotherapy] on my neck, in the base of my brain, and wow, it saved my life in my opinion” (403).

If money were no object, participants said they would undergo useful therapies more often. In particular, several participants expressed an interest in more frequent visits to massage therapists and acupuncturists. Others expressed a desire to trial new therapies that they had heard of by word of mouth or on social media. Some of these CAM modalities included zero-gravity and float tank therapies, stem cell therapies, and prolotherapy.

### 3.2 Approval and disapproval of CAM modalities

Our participants had undertaken a wide variety of CAM therapies. The majority of survey respondents had trialed dietary supplements (81%) and massage therapy (81%). The most common dietary supplement was magnesium (*N* = 8, or 32%), followed by B, C, and D vitamins, and fish oil (each *N* = 5, or 20%) (see Table 3). After dietary supplements and massage therapy, the most common CAM modalities were acupuncture (65%) and chiropractic therapy (65%). Mindfulness and meditation had been practiced by 61% of respondents. All other modalities were used by fewer than 50% of participants. For more information, see Table 4.

**TABLE 3:** Dietary supplements.

	*N* = 25 (%)
Magnesium	8 (32%)
B vitamins	5 (20%)
C vitamins	5 (20%)
D vitamins	5 (20%)
Fish oil	5 (20%)
CoQ10	2 (8%)
Multivitamin	2 (8%)
Zinc	2 (8%)

**TABLE 4:** Complementary and alternative medical (CAM) modalities trialed.

	Trialed	Found helpful	Found unhelpful
Dietary supplements^a^	25 (81%)	14 (56%)	7 (28%)
Massage therapy^c^	25 (81%)	21 (84%)	4 (16%)
Acupuncture^c^	20 (65%)	4 (20%)	13 (65%)
Chiropractic^c^	20 (65%)	7 (35%)	9 (45%)
Mindfulness^b^	19 (61%)	13 (68%)	5 (26%)
Cannabis/CBD^a^	15 (48%)	12 (80%)	2 (13%)
Relaxation^d^	11 (35%)	3 (27%)	3 (27%)
Aroma therapy^d^	11 (35%)	8 (73%)	3 (27%)
Yoga^d^	10 (32%)	4 (40%)	6 (60%)
Pain coping skills^d^	9 (29%)	6 (67%)	3 (33%)
Ultrasound^c^	8 (26%)	4 (50%)	2 (25%)
Pulse magnetic pads^c^	7 (23%)	5 (71%)	2 (29%)
Self-hypnotic relaxation^b^	5 (16%)	3 (60%)	2 (40%)
Hypnosis^b^	4 (13%)	2 (50%)	2 (50%)
Laser therapy^c^	4 (13%)	2 (50%)	1 (25%)
Low-energy neurofeedback system^d^	4 (13%)	1 (25%)	3 (75%)
Qigong^d^	4 (13%)	3 (75%)	0 (0%)

Modalities trialed by four or more participants.

^a^Nutritional.

^b^Psychological.

^c^Physical.

^d^Combination of psychological and physical.

#### 3.2.1 Most useful CAM modality

Overall, 58% of CAM modalities were considered helpful by survey respondents who had trialed them. They rated massage therapy (*N* = 21 or 84%), medical cannabis (*N* = 12 or 80%), and mindfulness (*N* = 13 or 68%) as some of the most useful CAM modalities for managing symptoms related to hEDS. For more information, see Table 4. In our qualitative data, interviewees described massage (*N* = 6, or 25%), dietary supplements (*N* = 5, 21%), mindfulness (*N* = 4, 17%), and cannabis/CBD (*N* = 4, 17%) as the most useful CAM modalities. For more information, see Table 5. Interviewees described using many different forms of cannabis, though many preferred not to use a smoked form of the drug. “I find that the best for me is topical versions of [cannabis] that are very helpful when I’m having muscle spasms and deep pain,” as one representative participant noted (721).

**TABLE 5:** Qualitative reporting of most useful complementary and alternative medical (CAM) modalities.

CAM modality	*N* = 24
Massage	6
Supplements	5
Mindfulness	4
Cannabis/CBD	4
Transcutaneous electric nerve stimulation (TENS)	3
Myofascial release	3
Acupuncture	2
Dry needling	2

Modalities that two or more interviewees rated as most useful.

#### 3.2.2 Least useful CAM modalities

Overall, 32 percent of CAM modalities were considered unhelpful or harmful by survey respondents who had trialed them. They rated acupuncture (*N* = 13, or 65%) as the least helpful modality for managing symptoms related to hEDS. For more information, see Table 4. In our qualitative data, interviewees described chiropractic therapy (*N* = 7, or 29%) and acupuncture (*N* = 5, 21%) as the least useful CAM modalities. One participant listed both of these modalities as equally unhelpful, stating that “the chiropractor just made [my symptoms] worse, and acupuncture was very painful but didn’t help anyway” (418). For more information, see Table 6.

**TABLE 6:** Qualitative reporting of least useful complementary and alternative medical (CAM) modalities.

CAM modality	*N* = 24
Chiropractor	7
Acupuncture	5
Dry needling	2
Laser therapy	2
Physical therapy	2
Ultrasound	2

Modalities that two or more interviewees rated as least useful.

### 3.3 Trust in CAM modalities and practitioners

Participants suggested an overall trust in CAM modalities and practitioners. They did not understand their research or vetting habits regarding CAM treatments to be significantly different from their habits regarding conventional treatments. Likewise, they did not understand their level of trust to differ between CAM and conventional healthcare providers. However, they found CAM practitioners to be more attentive to their subjective symptoms and experiences than conventional medical providers, and this affected their relationships in a positive manner. “I think [CAM practitioners] are more open to listening to what you have to say and your story and background” (983). Participants also described CAM providers as less skeptical of their hEDS diagnoses and medical histories: “I feel like [CAM practitioners] are not questioning you as much. Or it’s more just listening and then trying to respond to what you’re saying” (100). In general, this greater receptivity and lack of skepticism motivated participants’ overall trust in CAM practitioners.

Conversely, some participants (*N* = 8) expressed distrust in CAM practitioners. In general, this distrust seemed to center on the financial costs associated with certain CAM treatments. “I hate to call them criminals, but the people that pretend to be functional medicine [doctors] are charging thousands of dollars to give just basic nutritional advice” (403). One participant who expressed a high level of trust in CAM practitioners offered the following caveat with regard to her trust: “Some alternative providers have turned into total predators” (029).

Despite being driven to new therapies by desperation for symptoms relief, our participants described overall savvy consumption practices. While they felt CAM providers were often more attentive to their needs—and thus occasionally more trustworthy—they were aware and cautious of ulterior motives and unevidenced treatments. Many refused to pursue CAM treatments without conducting their own research on the modality beforehand.

#### 3.3.1 Disclosure of CAM use to medical providers

Of the 12 interviewees who stated whether they would disclose their CAM usage with medical providers, a majority (*N* = 9) said that they were comfortable doing so. However, some participants expressed a worry that clinicians would be dismissive or antagonistic to such treatments: “They’re going to look at you and be like, “Oh, no, [CAM practitioners] are crazy. They’re quacks!” People who I would trust the opinions of have been called frauds by [conventional medicine] providers” (400). In addition to this, using a CAM modality with an attached social stigma influenced whether participants felt comfortable discussing CAM with their medical provider: “I don’t openly always disclose the cannabis because […] you get a look. And then I know that will probably jade them for the rest of my treatment” (500).

#### 3.3.2 Skepticism of and injury from CAM

Attitudes toward CAM were generally positive. However, some participants (*N* = 5) described a critical skepticism of CAM therapies as such. In particular cases, participants were quite overt in their suspicion of the efficacy of certain CAM treatments: “I just closed my eyes and laid there while she did stuff around me with the tuning forks. It was supposed to balance my energy or something […] She goes “Well, I called in spirits to help balance you and that’s probably what you felt,” and I was like, “Okay, whatever”” (105).

A source of even greater critical skepticism among participants, however, arose during their descriptions of injuries that had resulted from CAM treatments. Typically, these participants (*N* = 12) ascribed their injuries to practitioners’ unfamiliarity with the physical issues associated with hypermobility. For instance, one woman was left with “really, really horrible” bruising after a cupping procedure (158). While some bruising may be expected from a cupping procedure, she stated, clinicians educated on hEDS would be aware that this population can incur long-lasting bruises from even minor traumas, due to tissue fragility. Several participants described being in pain after trialing chiropractic therapy, massage, and yoga. One participant complained that her chiropractor “did a whole lot more damage than help” and stated that she needed “years to recover from it” (983). Another interviewee described her concern with her acupuncturist, who “tried to get me to go off my seizure medications, and that was too out-there and too stupid to try […] She was really trying to convince me that all of my medications were bad for me and trying to take me off of life-saving medication” (552).

Additionally, participants characterized certain CAM treatments as inherently dangerous for the hEDS population. One participant described how the Alexander Technique—a CAM modality that focuses specifically on spinal posture—can result in various injuries for hypermobile patients: “The Alexander Technique has a very big focus on the head and neck, kind of leading the whole body, and if you have craniocervical instability, […] it’s really easy to cause subluxations” (692).

When risks and costs of CAM use were sufficiently low, several participants stated that the medically verifiable efficacy of such treatments was not necessarily their most critical feature. “Just because it doesn’t work, or maybe it’s a placebo effect, as long as it’s making someone feel better, I’m not going to judge them for it,” one woman said (506). She went on to explain that some of her acquaintances had trialed crystal healing, which she personally did not believe was an effective therapeutic option, but she thought it was reasonable for them to trial and even to continue the practice as they seemed to find benefit in it. Another interviewee said that she was unsure whether acupressure had been beneficial for her, but “it’s definitely interesting and relatively harmless,” so she thought it was reasonable to continue using it (400).

## 4 Discussion

Complementary and alternative medicine may be glossed as a group of diverse therapeutic practices and products that are not currently considered part of conventional, allopathic medicine. However, definitions of CAM paper over salient differences between modalities, and referring to CAM blanketly erases a heterogeneity of providers and treatments that is highly salient in understanding patients’ decision making. Previous studies have only reviewed a limited subset of CAM treatments, for instance, medical cannabis (3, 15), Traditional Chinese Medicine, and herbal medications (3). However, our participants listed a wide range of modalities that they considered complementary or alternative. Additionally, they identified a wide variety of symptoms they attempted to manage through CAM therapies, such as anxiety, dizziness, and insomnia, rather than just pain, which was the sole focus of previous work (3).

While we suggested specific therapies traditionally regarded as CAM, we ultimately left the task of defining the category to our participants. This technique allowed us to capture a much wider variety of practices and experiences than have other studies. For instance, the single other study focused on CAM and EDS only discussed three categories of CAM (3). With this limited definition, that study reported 56% of participants had made use of at least one of these CAM modalities. By expanding the modalities that we discussed beyond these three categories, we found that all of our interviewees had trialed at least one CAM therapy.

Our participants described a dissatisfaction with conventional means of symptom management, confirming an hypothesis posed by other researchers in this field (3), and they went on to describe a desperation for symptom relief, leading to their CAM use. However, our participants identified lack of insurance coverage and out-of-pocket costs as barriers to pursuing or continuing CAM. This qualitative finding is aligned with quantitative analysis regarding the out-of-pocket costs associated with CAM treatments (21). Interestingly, while a recent meta-analysis found that a perceived lack of side-effects or risks was often a motivating factor for pursuing CAM treatments (22), we did not find this motivation among our participants. Indeed, many of our participants reported injuries or negative experiences associated with certain CAM modalities. These injuries and negative experiences often served as a *de*-motivating factor in further pursuing these treatments.

Overall, our participants positively appraised massage therapy, which reflects early clinical findings that massage is a useful technique in providing relief for both physical pain and psychological stressors (23, 24). In addition to massage therapy, participants favored dietary supplements, with magnesium identified as the most used supplement by our participants. This positive assessment is less supported by the existing evidence. A recent review reported that there is insufficient evidence to support or refute the hypothesis that magnesium is an effective treatment for chronic pain (25). Regarding the use of cannabis for medicinal purposes, 48% of our participants had trialed some form of the substance, with a majority of them finding it helpful in providing symptom relief. This percentage of reported cannabis use is slightly higher than the percentage of use reported in an earlier study, which found its use in only 36.9% of an EDS sample population (3).

It should be recognized that many of the therapies trialed by the participants of this study have clinical evidence supporting their use in managing symptoms associated with hEDS. For instance, relaxation techniques, which were trialed by 35% of our survey respondents, have been shown to be successful in general pain control (26). Acupuncture, which was trialed by 65% of our survey respondents, was found to be effective in relieving facial pain, dental pain, and fibromyalgia-associated pain (27–29). Mind-body therapies (for example, meditation, mindfulness, relaxation, and biofeedback), which were trialed by a majority of our survey respondents, have likewise shown success in treating pain (30). Previous research has also found marijuana may be effective at reducing chronic pain symptoms in the short term (31), paralleling the anecdotal experiences of our participants.

However, while some clinical evidence supports the use of certain CAM modalities, there is no consensus on whether other CAM treatments should be recommended for a patient population such as those living with hEDS. For instance, while acupuncture has been shown to improve pain in certain parts of the body (see preceding paragraph), it is unclear whether it is useful in managing back and neck pain and headaches (32, 33), the primary types of pain affecting patients with hEDS. And as our interviews have demonstrated, some patients may find it more harmful than helpful. Such ambiguities in clinical effectiveness mean that while the utility of certain CAM modalities may be suggestive, their recommendation is not yet evidence-based and needs to be assessed relative to the individual patient’s capacities and thresholds.

Despite the overall positive appraisal of CAM treatments, our participants expressed varying levels of trust and skepticism of CAM providers. This finding affirms the conclusions of an earlier qualitative study, which examined the role that trust played in CAM clinics (34). That study found that relational trust between an individual patient and individual provider does not necessarily entail the patients’ trust in CAM as a whole. Thus, patients may express trust in one CAM provider but not in another, and patients’ risk assessment of proposed therapies also conditions their trust.

While the majority of our participants (75%) stated that they were comfortable discussing their CAM usage with their clinicians, previous studies have found that fewer than 40% were willing to disclose this information to physicians (35, 36). Moreover, a study of pediatric gastroenterology patients found that CAM use was discussed with clinicians in less than a quarter of cases (37). This discrepancy is likely explained by the additional context provided by our in-depth interviews, namely, that our participants’ willingness to disclose CAM use was based on pre-existing relational trust in their clinicians. They still readily acknowledged a fear and skepticism of unknown or disliked clinicians and a refusal to discuss these topics with them.

### 4.1 Limitations

Some bias may exist among our population for conventional medicine, as they were each recruited as patients at a conventional medicine clinic. However, it seems to be most common that patients engage in both conventional and CAM therapies, rather than only in CAM (36, 38). The demographics of our participants were representative of the population diagnosed with hEDS—the majority of our participants were White, female, and from the United States. However, as regional and cultural variation seem relevant to CAM use and preference, the demographics of our sample may also represent a limitation in the generalizability of our results. That said, our participants had a wide range of educational backgrounds and religious affiliations, factors that are also known to affect rates of CAM utilization (36, 38, 39).

## 5 Conclusion

Our study found that CAM therapies are a regular part of the health-seeking practices of patients with hEDS. Future research on this topic should determine whether similar trends in CAM usage exist in other disorders characterized by chronic pain and/or disorders that often feature difficult patient–provider relationships. While many of our participants believed these therapies were essential for their wellbeing, they also described injuries caused by them. Certain modalities, such as St. John’s wort, may have clinically relevant interactions with prescription medication; and other modalities, such as those involving joint manipulation (for example, chiropractic and massage therapy) may be inappropriate or need to be modified for patients with hypermobility.

It is therefore important for clinicians to foster therapeutic relationships with their patients, thereby allowing for clear and honest communication about CAM. Our participants expressed that when effective and compassionate clinical communication is present, they are willing to disclose their CAM usage to clinicians. However, studies have shown that clinicians only rarely ask about CAM and often feel uncomfortable broaching the topic (40, 41). The addition of a broad, non-judgmental question to inquire about CAM use may be appropriate, for example, “How else do you seek relief from your symptoms?” (42).

Our study shows that clinicians should be aware that many patients with chronic pain feel they have exhausted conventional treatments for managing their symptoms. CAM modalities that lessen patients’ symptoms (even only subjectively), improve their functionality, pose no health risk, and do not constitute a financial burden can be an important means of improving patient care. Openness to discussing empirically supported CAM modalities may benefit the patient–provider relationship, reduce symptoms burden, and establish open and honest communication regarding present and future CAM use. As patients continue to trial CAM treatments, it is important that clinicians be able and willing to provide their own critical evaluations and guidance on these health-seeking behaviors. Moreover, it is important for clinicians to recognize that their patients are navigating a complex interface of different healthcare systems and their attendant barriers in their pursuit of symptoms management. Future research is needed to determine whether similar trends in CAM usage exist in other disorders characterized by chronic pain.

## Data availability statement

The raw data supporting the conclusions of this article will be made available by the authors, without undue reservation.

## Ethics statement

The studies involving human participants were reviewed and approved by Indiana University School of Medicine IRB. The patients/participants provided their written informed consent to participate in this study.

## Author contributions

CH designed the study and collected the data. Both authors led the analysis and wrote and approved the manuscript for publication.
